# Unmet needs in squamous cell carcinoma of the lung: potential role for immunotherapy

**DOI:** 10.1007/s12032-014-0960-1

**Published:** 2014-04-19

**Authors:** Thomas E. Stinchcombe

**Affiliations:** Multi-disciplinary Thoracic Oncology Program, University of North Carolina UNC Chapel Hill, Physicians Office Bldg. CB# 7305, 170 Manning Drive, 3rd Floor, Chapel Hill, NC 27599-7305 USA

**Keywords:** Non-small cell lung cancer, Squamous cell carcinoma of the lung, Programmed cell death protein-1 (PD-1) pathway, Ipilimumab, Nivolumab, Immunotherapy

## Abstract

Squamous cell carcinoma of the lung accounts for 20–30 % of non-small cell lung cancers (NSCLC). Despite the differences in disease characteristics between squamous and non-squamous NSCLC, both have historically been treated similarly in the clinic. Recently approved drugs have revealed differences in activity and safety profiles across histologic subtypes and have applicability in treating non-squamous, but not typically squamous, NSCLC. Exploration of immune checkpoints—co-inhibitory molecules used to regulate immune responses—has resulted in novel immunotherapies designed to interrupt signaling through the cytotoxic T lymphocyte-associated antigen-4 or programmed cell death protein-1 pathways on lymphocytes. Modulation of these pathways can lead to restored antitumor immune responses, and preliminary evidence shows that agents targeting these pathways have activity in lung cancer, including squamous NSCLC.

## Background

The first-line treatment for advanced non-small cell lung cancer (NSCLC) depends on the tumor histology and the presence of epidermal growth factor receptor (*EGFR*) mutation or anaplastic lymphoma kinase (*ALK*) rearrangements. For NSCLC with mutated *EGFR* or positive for *ALK*, treatment with an EGFR tyrosine kinase inhibitor (e.g., erlotinib or afatinib) or ALK inhibitor (e.g., crizotinib), respectively, is recommended. These molecular abnormalities are more common in patients who have adenocarcinoma. For patients with non-squamous NSCLC without an actionable molecular abnormality, the standard therapy remains chemotherapy with or without bevacizumab. Patients with squamous histology have experienced a prohibitive rate of severe pulmonary hemorrhage when treated with bevacizumab, and bevacizumab is, therefore, contraindicated in patients with squamous histology tumors [1, 2]. The activity of pemetrexed is limited to non-squamous histology [3]. Therefore, additional options to combat squamous NSCLC are needed, and based on the preliminary findings to date, there is hope that immunotherapy will prove to be a novel therapeutic approach that is not limited by histologic subtype.

Newer data are providing a better understanding of how lung cancer can avoid immune detection or elimination and persist as clinical disease. As a result, immunotherapeutic approaches for the treatment of lung cancer treatment are rapidly evolving. These include novel agents, known as immune checkpoint inhibitors, which target mechanisms used by tumors for immune evasion. This immunotherapy approach differs from antigen-specific vaccines in that it targets the entire immune system. Immune checkpoints are co-inhibitory molecules that serve to balance or attenuate co-stimulatory pathways that lead to immune activation. In normal tissues, immune checkpoint pathways prevent overactivation of the immune system to protect self-tissues. In cancer, however, tumors can utilize immune checkpoint pathways to evade immune system recognition by expressing co-inhibitory molecules or their ligands [4–6]. Several of these co-inhibitory molecules [e.g., programmed cell death protein-1 (PD-1), its ligands PD-L1 and PD-L2, and cytotoxic T lymphocyte-associated antigen-4 (CTLA-4)] have been linked to tumoral immune escape. Immune checkpoint inhibitors targeting CTLA-4, PD-1, and PD-L1 have shown promising clinical activity in patients with melanoma and, more recently, clinical activity in NSCLC across histologic subtypes [7–15].

This article will review differences between the histologic and immunologic characteristics of squamous versus non-squamous NSCLC, provide a brief overview of the biology of immune checkpoint inhibitors, and discuss the potential future role of antibody-mediated immune modulation in squamous NSCLC based on clinical experience to date.

## Differences in histologic and immunologic characteristics between squamous and non-squamous NSCLC

Squamous and non-squamous NSCLC are recognized as distinct diseases, differing in terms of histologic and immunologic characteristics. Compared with non-squamous NSCLC, squamous NSCLC is associated with a higher likelihood of smoking, central tumor growth, and cavitation [16]. These differences between squamous and non-squamous NSCLC may influence the response to therapeutic agents, as exemplified by the differential profiles of targeted agents. Therefore, clinical trials are now starting to enroll patients by histologic type.

### Histologic characteristics

Well-differentiated squamous cell NSCLC is characterized by keratinization, intercellular bridges, and pearl formation [16, 17]. Tumor cells tend to be large with abundant dense cytoplasm, irregular hyperchromatic nuclei, and small nucleoli. For tumors without clearly differentiated morphology, analysis of p63 and thyroid transcription factor-1 (TTF-1) can be used to determine lineage [17]. Squamous cell carcinoma has a consistent histologic marker profile, namely diffuse expression of p63/CK5/6/34βE-12 and non-expression of TTF-1 [18]. In contrast, adenocarcinoma shows significant expression heterogeneity for all ‘squamous markers’; only diffuse TTF-1 expression is specific for adenocarcinoma. During attempts to refine histologic classification based on p63 expression, investigators found that TAp63, a p53-like tumor suppressor, was detected in 100 % of squamous cell carcinomas and in 31 % of adenocarcinomas. In contrast, expression of ΔNp63, an oncogene, was detected in 100 % of squamous cell carcinomas, but in only 3 % of adenocarcinomas [19].

### Immunologic characteristics

Many tumor antigens of interest in lung cancer, including melanoma-associated antigens A3 and A4 and NY-ESO-1, have more frequent expression in squamous tumors than in non-squamous tumors [20–23]. Furthermore, immunohistochemical analyses have shown that squamous NSCLC tumors have more extensive infiltration of CD8 + effector cells than do non-squamous tumors. However, the increased numbers of tumor-infiltrating CD8 + T cells did not correlate with a survival advantage, suggesting an impotent immune response, possibly due to an immunosuppressive tumor environment [24–26]. Genomic analysis of squamous cell carcinomas found that many samples exhibited inactivating mutations in the human leukocyte antigen-A class I major histocompatibility gene, which could lead to loss-of-function and reduced expression of tumor antigens, a possible immune-evading strategy [27].

## Biology of immune checkpoint inhibitors

### CTLA-4 blockade

The CTLA-4 receptor is expressed on T cells, and its interaction with ligands B7-1 (CD80) and B7-2 (CD86) is thought to attenuate T cell activation early in the activation process when the T cells are in the draining lymph nodes [28, 29]. CTLA-4 competes with the co-stimulatory receptor CD28 for B7 binding, preventing sustained T cell activation [30–32]. Blockade of CTLA-4 using monoclonal antibodies induces antitumor activity in murine cancer models, via both direct enhancement of T effector cells and concomitant inhibition of regulatory T cells [33–35].

### PD-1/PD-L1 blockade

Binding of the PD-1 receptor on T cells to its ligands, the most well characterized of which are PD-L1 and PD-L2, is another mechanism by which the immune system downregulates T cell activity; this pathway is thought to act later in the antitumor process, in the tumor microenvironment [36–38]. As evidence for this pathway’s role in immune evasion, PD-1 expression has been found on tumor-infiltrating T cells [39, 40], as has expression of PD-1 ligands on various tumor types, including squamous NSCLC [36, 41, 42]. In addition to T cells, B cells and natural killer cells can also express PD-1, which may reduce their antitumor activity [43].

PD-1:PD-L2 binding has higher affinity and is slightly different compared with PD-1:PD-L1 binding, although it is unclear whether these differences translate to different antitumor effects [44]. There is also evidence that PD-L1 may interact with CD80 (B7-1) expressed on T cells to suppress T cell activity [45, 46]. In preclinical models, antibody blockade of PD-1 or its ligands induces antitumor activity in murine cancer models through enhancement of T cell activity, which served as the basis for clinical development of PD-1 pathway-targeting agents [36, 47, 48].

## Preliminary clinical experience with immune checkpoint inhibitors in squamous NSCLC

### Ipilimumab

**Fig. 1:**
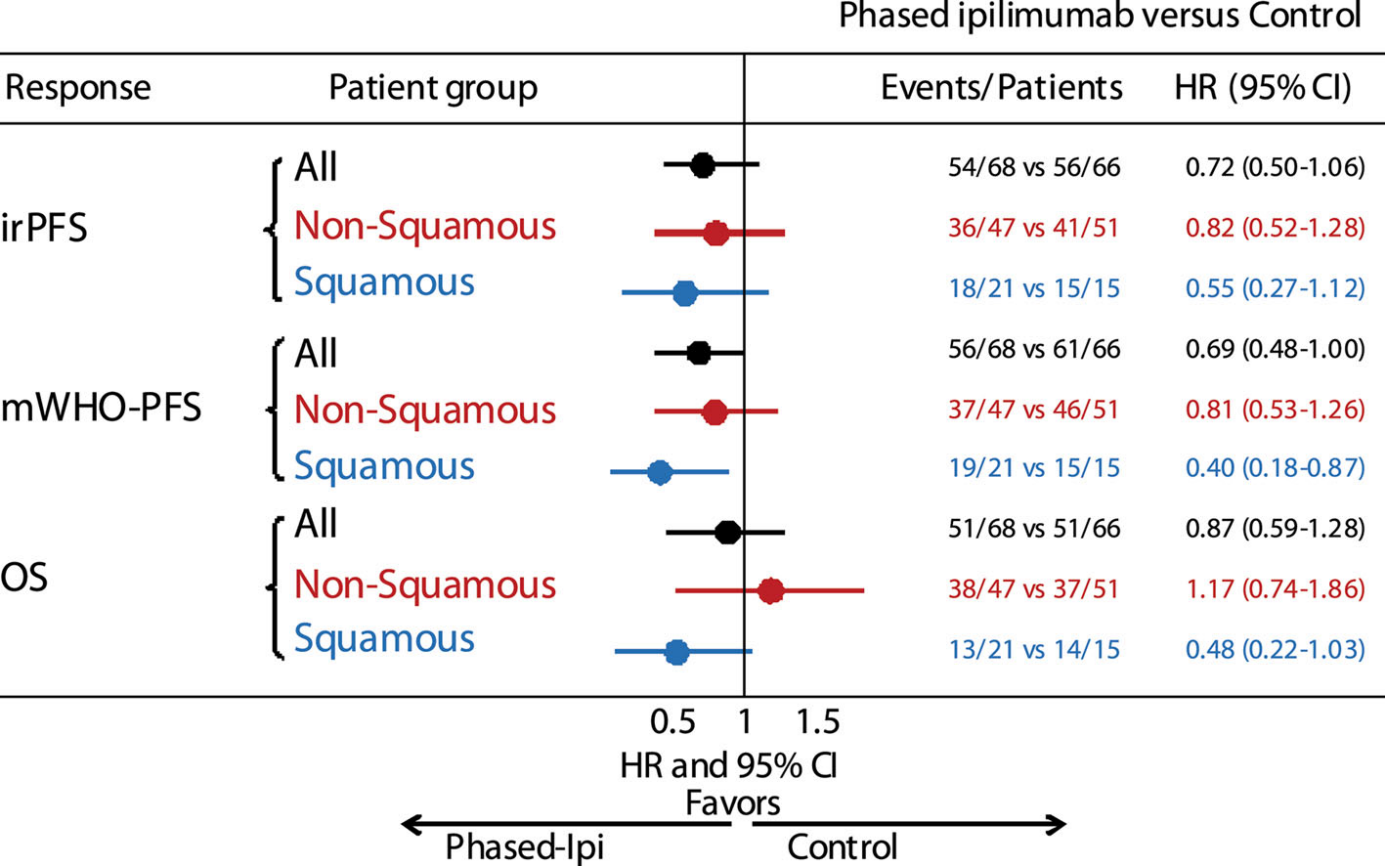
Clinical efficacy of phased ipilimumab + carboplatin/paclitaxel by histologic subtype in patients with NSCLC [49]. irPFS, progression-free survival (PFS) by modified WHO (mWHO) criteria and overall survival (OS) in the phase II randomized study of ipilimumab administered either in a phased schedule or currently with paclitaxel/carboplatin in patients with NSCLC, analyzed by histologic subtype. Comparison of the phased ipilimumab arm versus placebo arm. Phased ipilimumab plus paclitaxel/carboplatin appeared to have a greater effect on patients with squamous histology than those with non-squamous histology. The hazard ratio (HR) point estimates for irPFS, mWHO-PFS, and OS were significantly smaller with phased ipilimumab plus paclitaxel/carboplatin in the squamous population compared with the non-squamous population; however, small sample size warrants caution in interpretation. Reproduced with permission from Zielinski et al. [49]

Ipilimumab is an anti-CTLA-4 monoclonal antibody that binds to the CTLA-4 receptor, which restores CD28 signaling and allows immune activation to persist. In a randomized phase II study in previously untreated patients with NSCLC, the combination of ipilimumab with paclitaxel and carboplatin as a phased regimen (i.e., 2-dose chemotherapy lead-in) significantly improved immune-related progression-free survival (irPFS) and modified World Health Organization-PFS compared with chemotherapy alone (Table 1) [9]. The PFS advantage was not observed when ipilimumab was administered concurrently with chemotherapy (i.e., no chemotherapy lead-in); it is not clear whether these observations can be applied to other immune checkpoint inhibitors. Overall survival (OS) was longer in the phased ipilimumab treatment group, but the difference was not statistically significant. Subgroup analysis by histology indicated that the phased ipilimumab/chemotherapy regimen showed activity in both squamous and non-squamous NSCLC (Fig. 1). An ongoing phase III study (NCT01285609) is further evaluating the clinical efficacy of ipilimumab in combination with chemotherapy in patients with squamous NSCLC (Table 2).

**Table 1:** Clinical results of ipilimumab in combination with chemotherapy in patients with chemotherapy-naive advanced (Stage IIIB or IV) NSCLC [9]

	Phased ipilimumab	Concurrent ipilimumab	Chemotherapy control
Median time to immune-related progression	5.7 months	5.5 months	4.6 months
HR and *P* value versus control	HR = 0.72, 0.05	HR = 0.81, 0.13	
Median progression-free survival (WHO criteria)	5.1 months	4.1 months	4.2 months
HR and *P* value versus control	HR = 0.69, 0.02	HR = 0.88, 0.25	
OS, median	12.2 months	9.7 months	8.3 months
*P* value versus control	HR = 0.87, 0.23	HR = 0.99, 0.48	
OS at 1 year	50 %	42 %	39 %

*HR* hazard ratio, *OS* overall survival, *WHO* World Health Organization

**Table 2:** Ongoing phase II and III clinical trials of immune checkpoint inhibitors in NSCLC [50]

Target (trial identifier)	Trial phase	Trial stage (expected accrual)	Treatment setting	Clinical end points
Ant-CTLA-4				
Ipilimumab + paclitaxel/carboplatin versus placebo + paclitaxel/carboplatin (NCT01285609)	III	Recruiting (*n* = 920)	Stage IV/recurrent squamous NSCLC	Primary: OS secondary: OS, PFS, BORR
Anti-PD-1				
MK-3475 monotherapy versus docetaxel (NCT01905657)	II/III	Recruiting (*n* = 920)	Second-line, PD-L1-positive NSCLC	Primary: OS, PFS, safety Secondary: ORR, DOR
Nivolumab monotherapy (NCT01721759)	II	Ongoing (*n* = 100)	≥Third-line, advanced, or metastatic squamous NSCLC	Primary: IRC-assessed ORR Secondary: Investigator-assessed ORR
Nivolumab monotherapy after azacitidine + entinostat versus after oral azacitidine (epigenetic priming study) (NCT01928576)	II	Recruiting (*n* = 120)	≥Second-line,^a^ advanced, or metastatic NSCLC	Primary: response (progression-free at 32 weeks) Secondary: PFS, time to progression, OS, safety
Nivolumab monotherapy versus docetaxel (NCT01642004)	III	Ongoing (*n* = 264)	Second-line, advanced, or metastatic squamous NSCLC	Primary: ORR, OS secondary: PFS, PD-L1 biomarker, DOR, TTR, QOL
Nivolumab monotherapy versus docetaxel (NCT01673867)	III	Ongoing (*n* = 574)	Second-line, advanced, or metastatic non-squamous NSCLC	Primary: OS Secondary: ORR, PFS, PD-L1 expression, QOL
Anti-PD-L1				
MPDL3280A monotherapy (NCT01846416)	II	Recruiting (*n* = 130)	Advanced or metastatic PD-L1-positive NSCLC	Primary: investigator-assessed ORR Secondary: ORR, DOR, PFS, safety, PK
MPDL3280A monotherapy versus docetaxel (NCT01903993)	II	Recruiting (*n* = 180)	≥Second-line, advanced or metastatic NSCLC	Primary: OS secondary: ORR, PFS, safety, QOL

Trials enrolling patients with multiple tumor types, including NSCLC, are not listed

*BORR* best overall response rate, *DOR* duration of response, *IRC* independent radiology review committee, *NSCLC* non-small cell lung cancer, *ORR* objective response rate, *OS* overall survival, *PD*-*L1* programmed cell death protein-1 ligand-1, *PFS* progression-free survival, *PK* pharmacokinetics, *QOL* quality of life, *TTR* time to response

^a^Enrolled patients have ≤3 prior therapies

### MK-3475

MK-3475 is a humanized antibody against PD-1. MK-3475 and nivolumab (discussed below) both target PD-1 and are designed to inhibit PD-1 from binding its ligands, PD-L1 and PD-L2. In a phase I study, MK-3475 was administered at 10 mg/kg every 3 weeks to NSCLC patients previously treated with two systemic regimens [14]. Interim data on 38 patients showed an objective response rate (ORR) by Response Evaluation Criteria in Solid Tumors (RECIST) v1.1 criteria of 21 %, and responses were observed in both squamous and non-squamous histologic subtypes (Table 3). Rapid (within 9 weeks) and long duration of responses were reported.

**Table 3:** Interim phase I efficacy results of MK-3475 monotherapy (10 mg/kg) in evaluable patients with NSCLC [14]

ORR,^a^ *n/N* (%) [95 % CI]			Estimated median PFS, weeks (95 % CI)			Median OS, weeks (95 % CI)		
Squamous (*n* = 6)	Non-squamous (*n* = 26)	All NSCLC (*n* = 33)	Squamous (*n* = 6)	Non-squamous (*n* = 26)	All NSCLC (*n* = 33)	Squamous (*n* = 6)	Non-squamous (*n* = 26)	All NSCLC (*n* = 33)
2/6 (33) [4, 78]	4/26 (16) [4, 35]	7/33 (21) [9, 39]	15.2 (1.4, NR)	10.3 (7.6, 17)	9.7 (7.6, 17)	NR (2.7, NR)	35 (14, NR)	51 (14, NR)

*CI* confidence interval, *CR* complete response, *NSCLC* non-small cell lung cancer, *NR* not reached, *ORR* objective response rate, *OS* overall survival, *PFS* progression-free survival, *PR* partial response, *RECIST* response evaluation criteria in solid tumors

^a^ORR = [(CR + PR)/*n*] × 100; response rate per RECIST v1.1 was based on those patients who had ≥1 measurable lesion at baseline per central review. All responses were confirmed except for two

A phase I trial is currently recruiting patients with stage IIIB/IV NSCLC to evaluate combination therapy of MK-3475 with cisplatin/pemetrexed or carboplatin/paclitaxel (NCT01840579). Another phase I trial is assessing the safety and activity of MK-3475 monotherapy after platinum failure in patients with PD-L1-positive advanced NSCLC (NCT02007070). A phase II/III trial, also currently recruiting, will compare two doses of MK-3475 versus docetaxel in participants with PDL-1-positive NSCLC who have experienced disease progression after platinum-containing systemic therapy (NCT01905657) (Table 2).

### Nivolumab

Nivolumab is a fully human anti-PD-1 monoclonal antibody that binds to the PD-1 receptor, preventing it from engaging with its ligands. A phase I dose-escalating (0.1–10.0 mg/kg every 2 weeks) study of nivolumab initially reported results for 296 patients with advanced solid tumors, including 122 patients with NSCLC [11]. Most NSCLC patients had been previously treated; 94 % had received platinum-based chemotherapy and 34 % had received tyrosine kinase inhibitors. Objective (partial or complete) responses by RECIST v1.0 were reported in 14 of 76 evaluable NSCLC patients (18 %). The highest ORR (32 %) occurred with 3.0 mg/kg dosing. Six of 18 patients (33 %) with squamous NSCLC and 7 of 56 patients (12 %) with non-squamous NSCLC had a response. A longer-term interim analysis of 129 NSCLC patients enrolled in the study showed longer duration of responses and more sustained OS with nivolumab compared with previous reports of data from chemotherapy trials (Table 4). OS across all NSCLC patients was 42 % at 1 year and 24 % at 2 years [13]. Based on these findings, further investigation is being conducted with nivolumab (3.0 mg/kg) in patients with squamous NSCLC (NCT01642004) (Table 2).

**Table 4:** Interim phase I efficacy results of nivolumab monotherapy in evaluable patients with NSCLC [13]

Nivolumab dose (mg/kg)	ORR,^a^ * n*/*N* (%) [95 % CI]			Estimated median response duration, weeks (range)			Median OS, months (95 % CI)^b^		
	Squamous (*n* = 54)	Non-squamous (*n* = 74)	All NSCLC^c^ (*n* = 129)	Squamous (*n* = 54)	Non-squamous (*n* = 74)	All NSCLC^c^ (*n* = 129)	Squamous (*n* = 54)	Non-squamous (*n* = 74)	All NSCLC^c^ (*n* = 129)
1.0	0/15 (0)	1/18 (5.6) [0.1, 27.3]	1/33 (3.0) [0.1, 15.8]	0	63.9 (63.9, 63.9)	63.9 (63.9, 63.9)	8.0 (2.6, 13.3)	9.9 (5.6, 22.7)	9.2 (5.6, 11.1)
3.0^d^	4/18 (22.2) [6.4, 47.6]	5/19 (26.3) [9.1, 51.2]	9/37 (24.3) [11.8, 41.2]	NR (16.1, 133.9+)	74.0 (24.3, 74.0+)	NR (16.1+, 133.9+)	9.5 (6.7, NE)	18.2 (10.3, 18.2)	14.9 (9.5, NE)
10.0	5/21 (23.8) [8.2, 47.2]	7/37 (18.9) [8.0, 35.2]	12/59 (20.3) [11.0, 32.8]	83.1 (16.1, 117+)	NR (6.1+, 65.7+)	83.1 (6.1+, 117.1+)	10.5 (7.8, 12.5)	7.4 (4.6, 12.4)	9.2 (5.2, 12.4)
All doses	9/54 (16.7) [7.9, 29.3]	13/74 (17.6) [9.7, 28.2]	22/129 (17.1) [11.0, 24.7]	NR (16.1, 133.9+)	63.9 (6.1+, 74.0+)	74.0 (6.1+, 133.9+)	9.2 (7.3, 12.5)	10.1 (7.2, 13.7)	9.6 (7.8, 12.4)

*CI* confidence interval, *CR* complete response, *NSCLC* non-small cell lung cancer, *NE* not estimable, *NR* not reached, *ORR* objective response rate, *PR* partial response, *RECIST* response evaluation criteria in solid tumors

^a^ORR = ([CR + PR]/*n*) × 100; RECIST v1.0

^b^OS estimates after 1 year reflect censoring and shorter follow-up for patients enrolling later in the study

^c^One patient had unknown histologic type

^d^3.0 mg/kg dosing is being further evaluated in phase III trials

An ongoing phase I trial is evaluating nivolumab as a monotherapy or in various treatment combinations in patients with stage IIIB/IV NSCLC (NCT01454102). In the nivolumab plus chemotherapy arms, patients are randomized to receive nivolumab plus gemcitabine/cisplatin, plus either pemetrexed/cisplatin or carboplatin/paclitaxel, with safety as the primary assessment. Nivolumab is administered every 3 weeks until progression, and chemotherapy is given for four cycles at standard dosing. Interim results showed evidence of activity in patients with squamous NSCLC who received combination therapy; however, no firm conclusions can be drawn from this small phase I study [51]. This trial is also evaluating nivolumab in combination with a targeted agent (erlotinib or bevacizumab), nivolumab plus ipilimumab, nivolumab as switch maintenance monotherapy after platinum doublet, and nivolumab monotherapy in patients with asymptomatic brain metastases.

### BMS-936559

The fully human anti-PD-L1 antibody BMS-936559 was studied in a phase I trial of patients with advanced cancer, including 75 NSCLC patients [7]. The NSCLC patients experienced responses when treated with the 3.0 mg/kg or the 10.0 mg/kg dose. Overall, 5 of 49 patients with NSCLC had an objective response and these responses lasted for ≥24 weeks in 3 of these 5 patients. One of 13 patients with squamous histology and 4 of 36 patients with non-squamous histology had a response; reported ORRs were 8 and 11 %, respectively. Six additional patients, three with squamous and three with non-squamous histology, had stable disease lasting at least 24 weeks. At 6 months, the rates of PFS were 43 and 26 % for patients with squamous and non-squamous NSCLC, respectively.

### MPDL3280A

MPDL3280A is a human anti-PD-L1 monoclonal antibody containing an engineered immunoglobulin G Fc-domain designed to optimize efficacy and safety. A phase I dose-ranging study of MPDL3280A monotherapy in patients with locally advanced or metastatic NSCLC reported an initial ORR of 23 % (12 of 53 patients); three patients with squamous NSCLC and nine patients with non-squamous NSCLC had a response [15]. Additional patients who were not included in the ORR (RECIST v1.1) had delayed responses after apparent radiographic progression.

An additional phase I study, currently recruiting patients, will evaluate MPDL3280A in combination with carboplatin/paclitaxel, with carboplatin/pemetrexed, and with carboplatin/nab-paclitaxel in patients with advanced or metastatic NSCLC (NCT01633970). Two additional phase II studies in patients with advanced or metastatic NSCLC are ongoing. One trial is evaluating objective responses in patients with PD-L1-positive NSCLC receiving single-agent MPDL3280A therapy (NCT01846416). The other trial is evaluating OS and safety of MPDL3280A compared with docetaxel after platinum therapy failure (NCT01903993) (Table 2). A phase III trial of similar design, comparing MPDL3280A with docetaxel, is planned to start in early 2014 (NCT02008227).

## Ongoing trials of immunotherapy in squamous cell NSCLC

Table 2 lists ongoing phase II and III trials of immune checkpoint inhibitors in NSCLC, including three trials specifically in squamous cell carcinoma of the lung.

An ongoing double-blind placebo controlled phase III study (NCT01285609) is comparing carboplatin (area under the curve of six) and paclitaxel (175 mg/m^2^) every 3 weeks with ipilimumab or placebo. The primary endpoint is OS. Patients will receive ipilimumab or placebo in combination with chemotherapy in a phased schedule, similar to that used in the phase II study described above: ipilimumab 10 mg/kg once every 3 weeks for four doses and then every 12 weeks beginning at week 24.

NCT01721759 is a single arm, multicenter, phase II study of nivolumab monotherapy in patients with advanced or metastatic squamous NSCLC who have received at least two prior treatment regimens. The trial has completed its enrollment. The primary endpoint is ORR, as determined by an independent radiology review committee. An ongoing, randomized open-label phase III study is comparing ORR and OS with nivolumab monotherapy versus docetaxel in patients with squamous cell NSCLC who progressed on or after platinum-based chemotherapy (NCT01642004).

With the exception of one trial enrolling patients with non-squamous NSCLC (NCT01673867), the additional ongoing trials listed in Table 2 are enrolling NSCLC patients who are not segregated at enrollment by histologic type. However, reports of these clinical trials may present results based on squamous or non-squamous histology, as this is frequently a stratification factor.

## Potential role of biomarkers in lung cancer immunotherapy

Predictive biomarkers for checkpoint inhibitor therapy would help to select patients who are most likely to respond to this treatment. However, a definitive predictive biomarker for any of these agents has not been identified. Expression of the PD-L1 ligand on tumor cells is the most promising predictive biomarker candidate reported to date for PD-1 pathway-targeting agents, but data are very limited at present.

In an immunohistochemical analysis of pretreatment biopsy specimens from several tumor types, tumor cell-surface expression of PD-L1 was significantly correlated with an objective clinical response to nivolumab monotherapy in a dose-ranging phase I study [11]. Nine of 25 patients (36 %) with PD-L1-positive tumors had an objective response versus 0 of 17 patients with PD-L1-negative tumors. However, this was a posthoc analysis on a subset of patients and the investigators urged caution in interpreting the results. Data correlating anti-PD-1/PD-L1 agent activity with PD-L1 tumor status are starting to emerge. Although some associations between PD-L1-positive status and increased activity have been noted, findings of several studies, including some in lung cancer, have reported responses in patients with tumors considered negative for PD-L1 expression [11, 12, 14, 15, 52]. Additional research is needed to determine the role of PD-L1 as a potential predictive marker of response to anti-PD-1 antibody therapy.

## Discussion

Histology is now an important consideration for treatment selection in NSCLC. Currently, patients with squamous NSCLC have more limited treatment options compared with patients with NSCLC of non-squamous histology. Immunotherapy with checkpoint inhibitors has the promise of being effective in various subtypes of NSCLC, as it directly targets tumoral escape mechanisms. Preliminary evidence suggests that activity with these agents may not be restricted by specific tumor cell characteristics or histology, and these results indicate that this novel approach to treatment may increase treatment options available to patients with squamous NSCLC. The results of ongoing phase II and III clinical trials of these agents will more clearly determine the future role for immunotherapy in the management of lung cancer, particularly for squamous cell carcinoma of the lung.

## References

[1] Johnson DH, Fehrenbacher L, Novotny WF, Herbst RS, Nemunaitis JJ, Jablons DM (2004). Randomized phase II trial comparing bevacizumab plus carboplatin and paclitaxel with carboplatin and paclitaxel alone in previously untreated locally advanced or metastatic non-small-cell lung cancer. J Clin Oncol.

[2] Reck M, Von PJ, Zatloukal P, Ramlau R, Gorbounova V, Hirsh V (2009). Phase III trial of cisplatin plus gemcitabine with either placebo or bevacizumab as first-line therapy for nonsquamous non-small-cell lung cancer: AVAil. J Clin Oncol.

[3] Scagliotti G, Hanna N, Fossella F, Sugarman K, Blatter J, Peterson P (2009). The differential efficacy of pemetrexed according to NSCLC histology: a review of two Phase III studies. Oncologist..

[4] Chen L (2004). Co-inhibitory molecules of the B7-CD28 family in the control of T-cell immunity. Nat Rev Immunol.

[5] Nirschl CJ, Drake CG (2013). Molecular pathways: coexpression of immune checkpoint molecules: signaling pathways and implications for cancer immunotherapy. Clin Cancer Res.

[6] Pardoll DM (2012). The blockade of immune checkpoints in cancer immunotherapy. Nat Rev Cancer.

[7] Brahmer JR, Tykodi SS, Chow LQ, Hwu WJ, Topalian SL, Hwu P (2012). Safety and activity of anti-PD-L1 antibody in patients with advanced cancer. N Engl J Med.

[8] Hodi FS, O’Day SJ, McDermott DF, Weber RW, Sosman JA, Haanen JB (2010). Improved survival with ipilimumab in patients with metastatic melanoma. N Engl J Med.

[9] Lynch TJ, Bondarenko I, Luft A, Serwatowski P, Barlesi F, Chacko R (2012). Ipilimumab in combination with paclitaxel and carboplatin as first-line treatment in stage IIIB/IV non-small-cell lung cancer: results from a randomized, double-blind, multicenter phase II study. J Clin Oncol.

[10] Robert C, Thomas L, Bondarenko I, O’Day S, Weber J, Garbe C (2011). Ipilimumab plus dacarbazine for previously untreated metastatic melanoma. N Engl J Med.

[11] Topalian SL, Hodi FS, Brahmer JR, Gettinger SN, Smith DC, McDermott DF (2012). Safety, activity, and immune correlates of anti-PD-1 antibody in cancer. N Engl J Med.

[12] Wolchok JD, Kluger H, Callahan MK, Postow MA, Rizvi NA, Lesokhin AM (2013). Nivolumab plus ipilimumab in advanced melanoma. N Engl J Med.

[13] Brahmer JR, Horn L, Antonia SJ, Spigel D, Ghandi L, Sequist LV, et al. Nivolumab (anti-PD-1; BMS-936558; ONO-4538) in patients with non-small cell lung cancer (NSCLC): overall survival and long-term safety in a phase 1 trial. Abstract MO18.03. 2013. https://www.webges.com/cview/library/wclc/home. Accessed 2 Dec 2013.

[14] Balmanoukian A, Hamid O, Hui R, Gandhi L, Leighl N, Gubens MA, et al. Preliminary clinical safety and activity of MK-3475 monotherapy for the treatment of previously treated patients with non-small cell lung cancer (NSCLC). Abstract MO18.02. 2013. https://www.webges.com/cview/library/wclc/home. Accessed 2 Dec 2013.

[15] Horn L, Herbst RS, Spigel D, Gettinger SN, Gordon MS, Hollebecque A, et al. An analysis of the relationship of clinical activity to baseline EGFR status, PD-L1 expression and prior treatment history in patients with non-small cell lung cancer (NSCLC) following PD-L1 blockade with MPDL3280A (anti-PDL1). Abstract MO18.01. 2013. https://www.webges.com/cview/library/wclc/home. Accessed 2 Dec 2013.

[16] Oliver TG, Patel J Akerley W. Squamous non-small cell lung cancer as a distinct clinical entity. Am J Clin Oncol. 2013. http://journals.lww.com/amjclinicaloncology/Abstract/publishahead/Squamous_Non_Small_Cell_Lung_Cancer_as_a_Distinct.99354.aspx. 10.1097/COC.0b013e3182a0e850 25806712

[17] Drilon A, Rekhtman N, Ladanyi M, Paik P (2012). Squamous-cell carcinomas of the lung: emerging biology, controversies, and the promise of targeted therapy. Lancet Oncol..

[18] Rekhtman N, Ang DC, Sima CS, Travis WD, Moreira AL (2011). Immunohistochemical algorithm for differentiation of lung adenocarcinoma and squamous cell carcinoma based on large series of whole-tissue sections with validation in small specimens. Mod Pathol.

[19] Bishop JA, Teruya-Feldstein J, Westra WH, Pelosi G, Travis WD, Rekhtman N (2012). p40 (DeltaNp63) is superior to p63 for the diagnosis of pulmonary squamous cell carcinoma. Mod Pathol.

[20] Bolli M, Kocher T, Adamina M, Guller U, Dalquen P, Haas P (2002). Tissue microarray evaluation of Melanoma antigen E (MAGE) tumor-associated antigen expression: potential indications for specific immunotherapy and prognostic relevance in squamous cell lung carcinoma. Ann Surg.

[21] Kim SH, Lee S, Lee CH, Lee MK, Kim YD, Shin DH (2009). Expression of cancer-testis antigens MAGE-A3/6 and NY-ESO-1 in non-small-cell lung carcinomas and their relationship with immune cell infiltration. Lung.

[22] Shigematsu Y, Hanagiri T, Shiota H, Kuroda K, Baba T, Mizukami M (2010). Clinical significance of cancer/testis antigens expression in patients with non-small cell lung cancer. Lung Cancer.

[23] Yoshida N, Abe H, Ohkuri T, Wakita D, Sato M, Noguchi D (2006). Expression of the MAGE-A4 and NY-ESO-1 cancer-testis antigens and T cell infiltration in non-small cell lung carcinoma and their prognostic significance. Int J Oncol.

[24] Hiraoka K, Miyamoto M, Cho Y, Suzuoki M, Oshikiri T, Nakakubo Y (2006). Concurrent infiltration by CD8+ T cells and CD4+ T cells is a favourable prognostic factor in non-small-cell lung carcinoma. Br J Cancer.

[25] Ikeda S, Funakoshi N, Inagaki M, Shibata T (2006). Clinicopathologic roles of tumor-infiltrating lymphocytes and CD8-positive lymphocytes in lung cancer imprint smears in squamous cell carcinoma and adenocarcinoma. Acta Cytol.

[26] Suzuki K, Kachala SS, Kadota K, Shen R, Mo Q, Beer DG (2011). Prognostic immune markers in non-small cell lung cancer. Clin Cancer Res.

[27] Cancer Genome Atlas Research Network (2012). Comprehensive genomic characterization of squamous cell lung cancers. Nature.

[28] Tivol EA, Borriello F, Schweitzer AN, Lynch WP, Bluestone JA, Sharpe AH (1995). Loss of CTLA-4 leads to massive lymphoproliferation and fatal multiorgan tissue destruction, revealing a critical negative regulatory role of CTLA-4. Immunity.

[29] Waterhouse P, Penninger JM, Timms E, Wakeham A, Shahinian A, Lee KP (1995). Lymphoproliferative disorders with early lethality in mice deficient in Ctla-4. Science.

[30] Parry RV, Chemnitz JM, Frauwirth KA, Lanfranco AR, Braunstein I, Kobayashi SV (2005). CTLA-4 and PD-1 receptors inhibit T-cell activation by distinct mechanisms. Mol Cell Biol.

[31] Riley JL, Mao M, Kobayashi S, Biery M, Burchard J, Cavet G (2002). Modulation of TCR-induced transcriptional profiles by ligation of CD28, ICOS, and CTLA-4 receptors. Proc Natl Acad Sci USA.

[32] Rudd CE, Taylor A, Schneider H (2009). CD28 and CTLA-4 coreceptor expression and signal transduction. Immunol Rev.

[33] Leach DR, Krummel MF, Allison JP (1996). Enhancement of antitumor immunity by CTLA-4 blockade. Science.

[34] Peggs KS, Quezada SA, Chambers CA, Korman AJ, Allison JP (2009). Blockade of CTLA-4 on both effector and regulatory T cell compartments contributes to the antitumor activity of anti-CTLA-4 antibodies. J Exp Med.

[35] Wing K, Onishi Y, Prieto-Martin P, Yamaguchi T, Miyara M, Fehervari Z (2008). CTLA-4 control over Foxp3+ regulatory T cell function. Science.

[36] Dong H, Strome SE, Salomao DR, Tamura H, Hirano F, Flies DB (2002). Tumor-associated B7-H1 promotes T-cell apoptosis: a potential mechanism of immune evasion. Nat Med.

[37] Freeman GJ, Long AJ, Iwai Y, Bourque K, Chernova T, Nishimura H (2000). Engagement of the PD-1 immunoinhibitory receptor by a novel B7 family member leads to negative regulation of lymphocyte activation. J Exp Med.

[38] Nishimura H, Nose M, Hiai H, Minato N, Honjo T (1999). Development of lupus-like autoimmune diseases by disruption of the PD-1 gene encoding an ITIM motif-carrying immunoreceptor. Immunity.

[39] Ahmadzadeh M, Johnson LA, Heemskerk B, Wunderlich JR, Dudley ME, White DE (2009). Tumor antigen-specific CD8 T cells infiltrating the tumor express high levels of PD-1 and are functionally impaired. Blood.

[40] Sfanos KS, Bruno TC, Meeker AK, De Marzo AM, Isaacs WB, Drake CG (2009). Human prostate-infiltrating CD8+ T lymphocytes are oligoclonal and PD-1+. Prostate.

[41] Boland JM, Kwon ED, Harrington SM, Wampfler JA, Tang H, Yang P (2013). Tumor B7-H1 and B7-H3 expression in squamous cell carcinoma of the lung. Clin Lung Cancer..

[42] Zou W, Chen L (2008). Inhibitory B7-family molecules in the tumour microenvironment. Nat Rev Immunol.

[43] Keir ME, Butte MJ, Freeman GJ, Sharpe AH (2008). PD-1 and its ligands in tolerance and immunity. Annu Rev Immunol.

[44] Rozali EN, Hato SV, Robinson BW, Lake RA, Lesterhuis WJ (2012). Programmed death ligand 2 in cancer-induced immune suppression. Clin Dev Immunol..

[45] Butte MJ, Keir ME, Phamduy TB, Sharpe AH, Freeman GJ (2007). Programmed death-1 ligand 1 interacts specifically with the B7-1 costimulatory molecule to inhibit T cell responses. Immunity.

[46] Park JJ, Omiya R, Matsumura Y, Sakoda Y, Kuramasu A, Augustine MM (2010). B7-H1/CD80 interaction is required for the induction and maintenance of peripheral T-cell tolerance. Blood.

[47] Blank C, Kuball J, Voelkl S, Wiendl H, Becker B, Walter B (2006). Blockade of PD-L1 (B7-H1) augments human tumor-specific T cell responses in vitro. Int J Cancer.

[48] Iwai Y, Ishida M, Tanaka Y, Okazaki T, Honjo T, Minato N (2002). Involvement of PD-L1 on tumor cells in the escape from host immune system and tumor immunotherapy by PD-L1 blockade. Proc Natl Acad Sci USA.

[49] Zielinski C, Knapp S, Mascaux C, Hirsch F (2013). Rationale for targeting the immune system through checkpoint molecule blockade in the treatment of non-small-cell lung cancer. Ann Oncol.

[50] ClinicalTrials.gov. 2013. http://clinicaltrials.gov. Accessed 3 Dec 2013.

[51] Rizvi NA, Antonia SJ, Chow LQM, Brahmer JR, Juergens RA, Borghaei H, et al. A phase I study of nivolumab (anti-PD-1; BMS-936558, ONO-4538) plus platinum-based doublet chemotherapy (PT-doublet) in chemotherapy-naive non-small cell lung cancer (NSCLC) patients (pts). Abstract 8072. 2013. http://meetinglibrary.asco.org/content/84225. Accessed 2 Dec 2013.

[52] Antonia SJ, Grosso JF, Horak CE, Harbison CT, Kurland JF, Inzunza HD, et al. Association of tumor PD-L1 expression and immune biomarkers with clinical activity in patients with non-small cell lung cancer (NSCLC) treated with nivolumab (Anti-PD-1; BMS-936558; ONO-4538). Abstract P2.11-035. 2013. https://www.webges.com/cview/library/wclc/home. Accessed 2 Dec 2013.

